# Chronic patient care at North West Province clinics

**DOI:** 10.4102/phcfm.v1i1.8

**Published:** 2009-04-21

**Authors:** Claire van Deventer, Ian Couper, Nontsikelelo Sondzaba

**Affiliations:** 1 Department of Family Medicine, University of the Witwatersrand, South Africa; 2 Centre for Rural Health, University of the Witwatersrand, South Africa

**Keywords:** chronic illnesses, monthly assessment, control, use of Essential Drugs List and Standard Treatment Guidelines

## Abstract

**Introduction:**

Chronic illnesses are a significant burden to the health services in South Africa. There is a specific national health plan whereby chronically ill patients who are acceptably controlled should be managed at clinic level. The perception has emerged that the management of primary care has not been optimal in the Southern District of the North West Province. This provided the motivation to initiate this research, namely consideration of chronic patient care at clinics in the North West Province of South Africa.

**Method:**

A cross-sectional descriptive study was carried out at four randomly selected clinics covering four sub-districts in the Southern District (North West Province). This was done using charts and registers at the clinics. Inclusion criteria were patients older than 18, and presenting with the following chronic illnesses: asthma/chronic obstructive airways disease (COAD), hypertension, diabetes and epilepsy. The major focus areas were the regular assessment of the patients, the level of control of the illness and the use of the Essential Drugs List and Standard Treatment Guidelines (EDL/STG).

**Results:**

In the cases of all the chronic illnesses it was found that regular assessments were poorly done, with asthma (peak flow measurements) being the most poorly done. Control was generally less than 50% for all the illnesses, although the EDL was followed fairly well by the personnel at the clinics.

**Conclusion:**

In the light of the burden of chronic illness the results give cause for great concern about the quality of care for chronically ill patients, and reasons were sought for some of the poor results. A subsequent decision was taken to carry out comprehensive quality improvement projects on each of the illnesses over the following five years.

## INTRODUCTION

After 1994 the Department of Health reconfigured the country's health services to include free clinic services and free access to health for pregnant women and children under six years.^1^ The government integrated primary care in the so-called supermarket approach to patient care.^2^

The down referral from the district hospitals to the community resources was also mapped out in the National Health Plan, and chronically ill patients who were not experiencing complications or needing specialised treatment were to be down-referred to their nearest clinics and health centres, where the visiting doctor would reassess them six monthly and the clinic sister would supply monthly medication as well as carry out basic screening tests.^1^

In this area there is a very large number of patients who are chronically ill. This was confirmed in a report to the National Directorate of Chronic Illness and Rehabilitation in July 2006. Annual statistics for 2005/2006 from the Southern District confirmed this.^3^

It has been suggested that the care of chronically ill people is often not optimal at clinics and that they then incorrectly access hospitals after-hours, in particular, in efforts to access what they perceive to be better care.

### Diabetes (NIDDM)

In South Africa, an estimated two to three million people are affected with diabetes mellitus (DM), more than one million of whom are undiagnosed. During the period 1990-2000 an increase of 30% in the prevalence of diabetes was reported in Africa, mostly due to a change of lifestyle and an increase in obesity.^4^

Hypertension (H/t) is a common co-morbidity to DM in South Africa and contributes significantly to morbidity in diabetes.^5–9^ It is therefore very important to optimise the care of diabetic and hypertensive patients and to persist in the maintenance of care of the highest standard. The bulk of this care rests on the primary care system and in particular the care provided by clinics.

### Hypertension

Hypertension has been targeted as a priority disease by the Reconstruction and Development Programme^10^ as well as by the National Department of Health.^11^ Recently, in Limpopo Province, a high prevalence of hypertension with poor levels of control was found amongst adults, as reported by the Demographic and Health Survey (2003).^12^ In Sub-Saharan Africa, in 2006, the prevalence was estimated at 10–20 million, out of 650 million people.^13^

### Asthma

**FIGURE 1:**
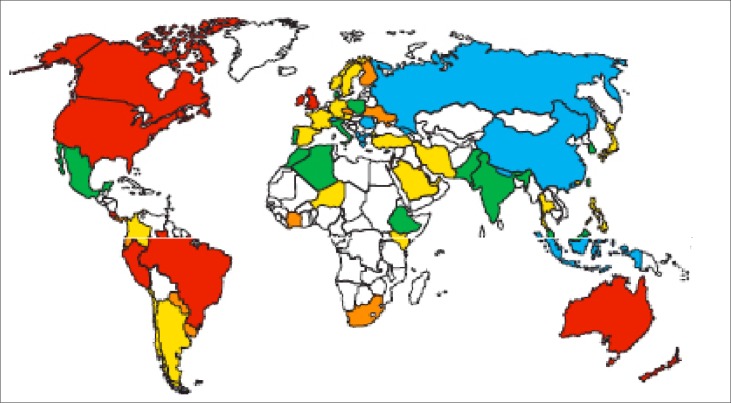
World map of the prevalence of asthma - taken from the GINA Burden of Asthma report (2007)13

A world map showing the prevalence of asthma, taken from the GINA Burden of Asthma report (2007)^14^ is shown in Figure 1. In this map, South Africa lies within the orange area (7.5–10% prevalence) and, according to the GINA report, has a proportion of 8.1% asthmatics in the population.

### Epilepsy

According to a US statistical survey,^15^ an estimated 375,851 out of 44,458,470 South Africans (about 0.8% of the population) have been diagnosed with epilepsy, as compared to in the USA (0.07%), Zimbabwe (0.24%) and Australia (0.8%). This is therefore not an illness that occurs in the large proportions that hypertension or diabetes do, but it does have a significant impact on the quality of life of patients (e.g. the ability to work, complete education, drive a car or use heavy machinery, amongst others).

A summary of chronic illnesses in the four subdistricts of the Southern District is presented in Table 1.

**TABLE 1:** Chronic illnesses in the subdistricts of the Southern District. District Health Information Systems (DHIS) June – December, 2005. Southern district.

DISTRICT	H/T	NIDDM	ASTHMA	EPILEPSY
Maquassie Hills	3707	3319	4078	3764
Klerksdorp	136407	18239	13962	15543
Potchefstroom	4961	318	363	6190
Ventersdorp	2231	215	252	220
Klerksdorp/Tshepong/Potchefstroom complex	10467	3803	1537	1293

### Aim and objectives

The overall aim of this study was to understand and evaluate the care of chronically ill patients at clinics in a district in the North West Province.

Specific objectives were the following:

- To investigate the prevalence of chronic diseases seen at the sampled clinics.
- To evaluate chronic disease drug management at the research clinics, using the Essential Drugs List and Standard Treatment Guidelines (EDL/STGs) as the gold standard.
- To explore the feelings and experiences of chronically ill patients at clinics regarding the management of their illnesses.

## METHOD

**Research site:** The research was carried out in four randomly selected clinics (Maquassie Hills, Ventersdorp, Matlosana and Potchefstroom) in the Southern District, North West Province, South Africa.

### Study design

This was a descriptive cross-sectional study involving the use of charts and records. Two data collection methods were used: focus groups for the qualitative data, and chart and record reviews for the quantitative data. Only the quantitative findings will be discussed in this article.

### Inclusion criteria

Patients with the following conditions were included: Patients above 18 years who were stable and visiting the clinic for follow-up care and patients having the following four common diseases, separately or in combination; asthma or chronic obstructive airways disease, NIDDM, hypertension or epilepsy.

### Exclusion criteria

Patients who were younger than 18 or presented with acute attacks needing hospitalisation were excluded.

## RESULTS

### General

The number of records accessed were: 695 hypertension, 136 epilepsy, 129 diabetes and 57 asthma.

Hypertension is by far the most prevalent chronic illness: 68% of the chronic patients assessed at the clinics were hypertensives. The predominance of females reflects 80.8% of the general clinic population with hypersensitivity. Females comprised 60.2% of diabetics, 70.2% of asthmatics and 44.8% of epileptics.

**FIGURE 2:**
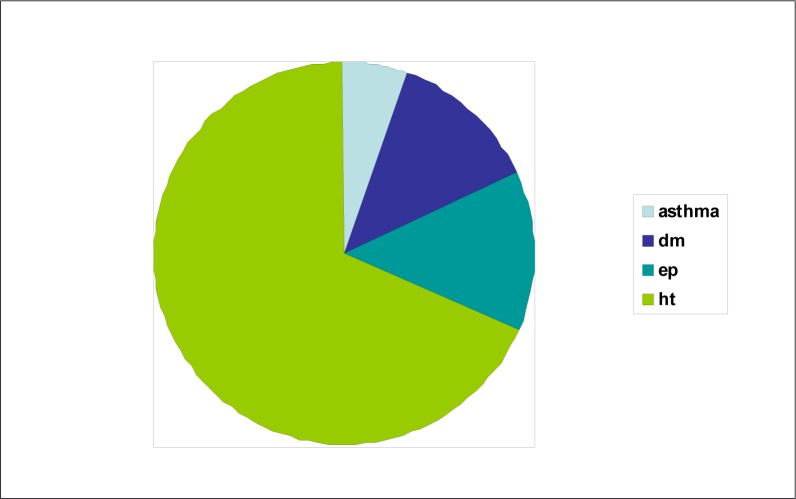
Proportion of the chronic illnesses: asthma, diabetes mellitus, epilepsy, hypertension

**FIGURE 3:**
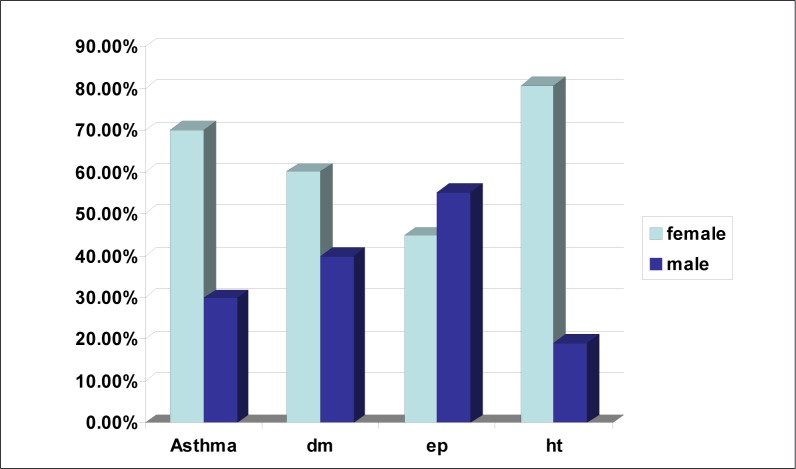
Sex prevalence in chronic illness

**FIGURE 4:**
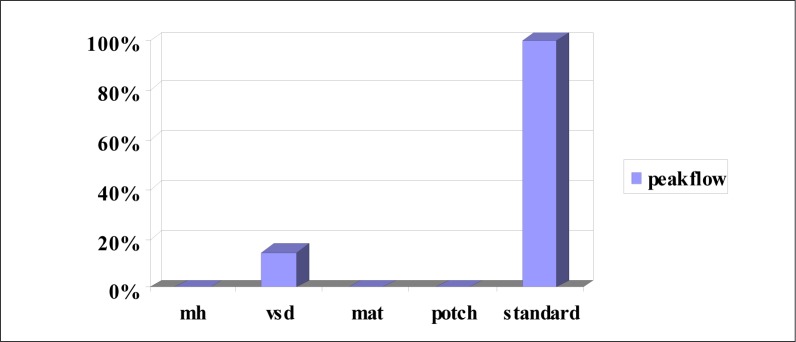
Asthma assessment at four clinics in South Africa (Maquassie Hills, Ventersdorp, Matlosana; Potchefstroom)

## CHRONIC CONDITIONS

### Asthma

**Monthly assessment:** Peak flow was recorded at all the clinics for an average of 3.5% of asthmatics only. All the outcomes were abnormal, and all of these were recorded at only one clinic.

**Control of illness:** It was not possible to measure the level of asthmatics with controlled asthma as the assessment (rate of peak flow readings) was so poorly done. The Standard Treatment Guidelines were followed in 75% of cases, whereby the most common prescriptions were the following:

- Salbutamol and beclomethazone (31.6%)
- Salbutamol only (21.1%)
- Salbutamol plus beclomethazone plus theophyllin (21.1%)

It was calculated that 2.5% of patients were using prednisone daily, in combination with other drugs. Theophyllin alone was given at two clinics: Matlosana (15.8%) and Maquassie Hills (20%).

There was no clear differentiation between COAD and asthma. This indicates that accurate diagnoses are lacking, as many patients were elderly and would therefore more probably have COAD rather than asthma.

### Diabetes

**Monthly assessment:** Urine testing was seldom done and a recorded body mass index (BMI) was not found for any patients. There was no screening for cholesterol and no health education documented in the files. No target organ disease was mentioned, e.g. eye or foot tests, and the HB1Ac blood test was not done for any of the patients. There was no documentation of health education given.

The blood glucose taken was a random specimen and was documented for an average of 53.8% of cases at all clinics.

**Control of illness**: Diabetes control was less than 20% at all the sites. The STG was followed in only 64% of patients. The most common prescriptions were:

- Metformin and gliclazide (23.3%)
- Metformin only (17.8%)
- Actraphane only (17.8%)
- Glibenclamide was very seldom used

**FIGURE 6:**
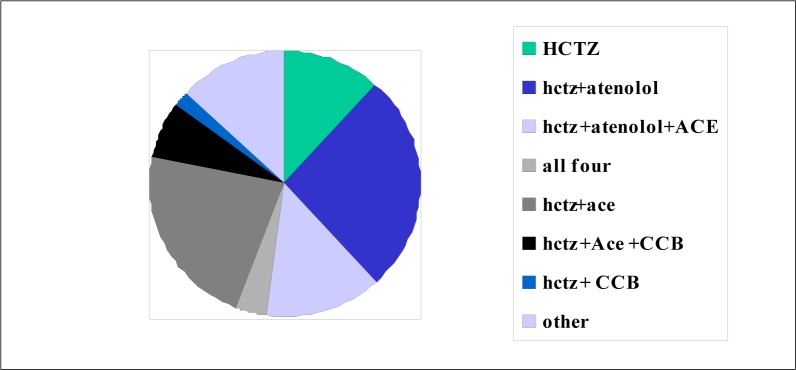
Control of chronic illness at four clinics in South Africa

There were a small number of patients on protophane only, metformin and actraphane, and maximum oral drugs plus actraphane. There were 71.3% of diabetic patients also using antihypertensives, with only 7% on the newer regimen (indapamide, perindopril and amlodipine). Please refer to Figure 6 for further information.

### Epilepsy

**Monthly assessment**: Of the 136 epileptic patients assessed across the four clinics 35 (29.7%) had had therapeutic blood levels recorded in the past year. The blood levels were normal in 40% of cases. The others were all sub-therapeutic.

**FIGURE 5:**
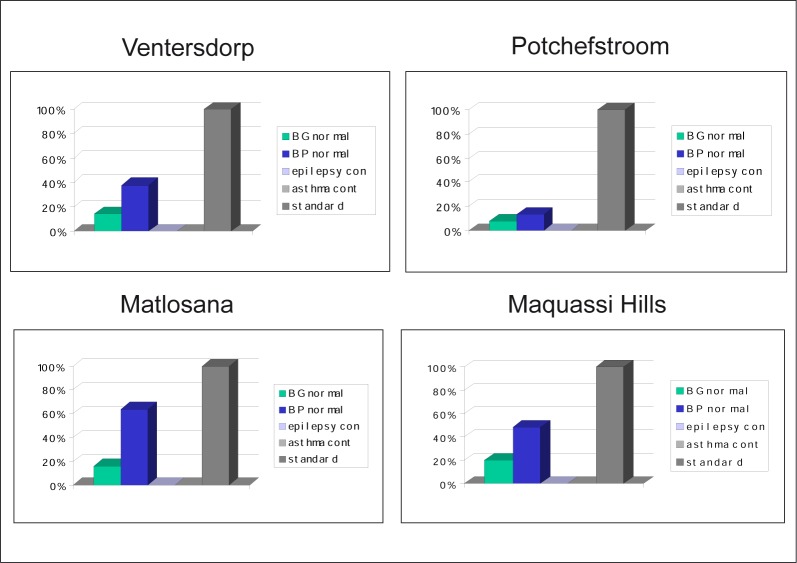
Control of chronic illness at four clinics in South Africa

The number of reported fits that had been documented was 65 (47.8%). The number of patients having more than two fits per month was 3 (4%), and those having less than 2, or none at all, was 62 (96%). Please refer to Figure 5 for further information.

**Control of illness**: Control of epileptic symptoms seemed to be good but information was limited. The STG was followed in 86.9% of cases, with 91% of patients on monotherapy. Ten patients were still having the controlled-release carbamazepine prescribed three times daily instead of twice daily.

### Hypertension

**Monthly assessment:** Monthly assessment, in terms of the completeness of records regarding monthly blood pressure monitoring, differed from clinic to clinic.

**Control of illness**: Control of hypertension varied between sites, from 16% to 60%. Please refer to Figure 5 for further information. STG was followed in 85% of cases, if one takes into consideration the new hypertension guidelines. The most common prescriptions were:

- Hydrochlorthiazide (HCTZ) and atenolol: 185 (26.7%)
- HCTZ + angiotensin converting inhibitor (ACE): 154 (22%)
- HCTZ + atenolol plus ACE: 96 (13.8%)
- Seven patients were still incorrectly on methyldopa

Calcium channel blocker (CCB) dosages were commonly 30 mg nifedipine: 53 (7.7%), 60 mg nifedipine: 17 (2.5%), while only three patients were on 90 mg nifedipine (0.4%). Enalapril was the most often used ACE inhibitor, as opposed to perindopril. Table 2 summarises the most pertinent findings as presented above.

**TABLE 2:** Combined results

	ASTHMA N = 57	DIABETES N = 129	EPILEPSY N = 136	HYPERTENSION N = 695
Sex	F = 70.2% M = 29.8%	F = 60.2% M = 39.8%	F = 44.8% M = 54.5%	F = 80.8% M = 17.9%
Age	65% are older than 50	70% are older than 50	41.9% are older than 50	70% are 50 or older
Screening done	3.5%	53.8%	25.7%	72.7%
Screening normal	0%	50%	40%	52.6%
STG followed	75%	64%	86.9%, with 91% on monotherapy	85%

The predominance of female patients is reflected here. Most of the patients, except epileptics, are older than 50.

Screening was very poorly carried out at all the clinics for asthmatics and epileptic patients. The monthly monitoring of diabetics was better than the above but still inadequate for good ongoing illness management.

The indicators of control in all four chronic illnesses warn that more than half of all chronically ill patients in the clinics studied are not having their diseases controlled.

## DISCUSSION

### Regular assessment of chronic patients

The regular assessment of vital signs is imperative for understanding and controlling an individual's illness. As there is (or should be) a monthly patient encounter, and even if only the basic tests are done at every clinic visit, this in itself is only a snapshot on a particular day. If the most basic screening is not being well done, that snapshot does not exist. It was heartening to find that Western province has been contending with similar difficulties. Steyn et al. found that 33% of the hypertensives had an acceptable blood pressure and only 42% of diabetics had below 11.1 non-fasting blood levels.^16^

The condition most poorly screened for is asthma. In spite of peak flow meters being available, there were no indications of this measurement being carried out and no documentation of symptoms or signs in the files to indicate whether these patients had asthma, as opposed to COAD, and whether they were persistent, mild, moderate or severe asthmatics. There were also no records regarding smoking in any of the files.

Epilepsy screening was done erratically and not according to the national guidelines, which stipulate that an annual blood therapeutic level should be checked in all epileptics.

It was of concern to see that only 40% of diabetics were being screened, as this is the condition with the most aggressive negative outcomes, e.g. renal failure, myocardial infarct, loss of vision and amputations.

### Following STG

The EDL/STG is a very simple guideline to follow. However, during the period that this study was carried out there was a significant factor that led to confusion. The EDL and the newer national hypertension guidelines (2006) differ in significant ways and in spite of awareness of and training in this area, confusion has persisted.

A positive finding was that the majority of epilepsy patients are on monotherapy at the clinics.

The diabetic STGs were influenced by down-referrals from local hospitals where non-EDL regimens were commonly being followed. Actraphane and metformin was a fairly common drug combination, but it is not supported by the primary care EDL and STG.

An alarming finding was the large percentage of diabetics with hypertension, and no plan had yet been devised to apply an optimal regime for this group, e.g. one including drugs as reported in the ASCOT study.^17, 18^

For all four conditions it was found that even where the required screening was being done the results were extremely poor. For all the conditions, good control of the illnesses was around 50% or less. In other words, health services in the Southern District are not managing to assist about half of their chronically ill patients to adequately treat their illnesses.

### Conclusion

The care of chronically ill patients in the four subdistricts in the Southern District of the North West Province that were surveyed was found to be less than optimal. It is recommended that a cycle of quality improvement be initiated per chronic illness in this district over the next five years, and that the research findings could assist in creating a sound basis from which to improve the situation.

## References

[1] South African Department of Health A National Health Plan for South Africa. Pretoria: Government Printers; 2003.

[2] South African Department of Health Health Monitoring and Evaluation. Health Goals, Objectives and Indicators. Pretoria: Goverment Printers; 2003.

[3] Chronic Illness Statistics Southern District, 2004 Presentation to the National Chronic Diseases Directorate, Potchefstroom, June 2006.

[4] Grobler F. Diabetes on the increase in Africa. Mail & Guardian [serial online]. 2002 [cited 2002 Aug 21]. Available from: http://www.mg.co.za/Content/13.jsp?a=37&0=7675

[5] Preston C. International Diabetes Federation: Raising public awareness around the World. WHO World Diab Newsletter. 1998;4:5–6.

[6] Westaway MS, Oosthuizen H, Van Zyl D. Health well-being and treatment satisfaction: Validating the Diabetes Treatment Satisfaction Questionnaire (DTSQ) for black and white South African diabetic patients Pretoria: Medical Research Council; 2002.

[7] Levitt NS, Steyn K, Daniels AD, Patel M, Lombard C, Zwarenstein M. Lack of impact of a national diabetes guideline based intervention package on glycaemic and blood pressure control at primary care level in Cape Town. J Endocrin Metab Diab S Afr. 2002;7:21.

[8] Saaddine JB, Engelgau MM, Beckles GL, Gregg EW, Thompson TJ & Narayan KM. A diabetes report card for the United States: Quality of care in the 1990s. Ann Intern Med. 2002;136:565–574. 11955024 10.7326/0003-4819-136-8-200204160-00005

[9] Kalk WJ, Pick WM, Sayed AR. Diabetes mortality in South Africa. S Afr Med J. 1998;88:1259–1262.

[10] South African Department of Health Hypertension: National Programme for Control and Management at Primary Level. Pretoria: Governemtn Printers; 1998.

[11] South African Department of Health, Medical Research Council and Macro International South African Demographic and Health Survey 1998. Preliminary Report. Pretoria: Government Printers; 1999.

[12] Igumbor EU, Bradshaw D, Laubscher R. Mortality Profile from Registered Deaths for Limpopo Province, South Africa 1997–2001 Cape Town: University of Venda/South African Medical Research Council; 2003.

[13] Medscape CME Medscape cardiology. 2006;10(1):1–9.

[14] Masoli M, Fabian D, Holt S, Beasley R. Global Initiative for asthma (GINA) program: The global burden of asthma: Executive summary of the GINA Dissemination Committee report. Allergy. 2004;59:469–478. 15080825 10.1111/j.1398-9995.2004.00526.x

[15] US Census Bureau 2004 International Database.

[16] Steyn K, Levitt NS, Patel M, et al Hypertension and diabetes: Poor care for patients at community health centres. SAMJ. 2008;98(8):618–622. 18928041

[17] Marshall SM, Flyvbjerg A. Prevention and early detection of vascular complications of diabetes. BMJ. 2006;333:4758. 10.1136/bmj.38922.650521.80 PMC1557968 16946335

[18] Statins for primary prevention in type 2 diabetes Drug and Therapeutics Bulletin. 2006;44:57–60. 10.1136/dtb.2006.44857 16903486

